# Actin Dynamics Regulate Multiple Endosomal Steps during Kaposi's Sarcoma-Associated Herpesvirus Entry and Trafficking in Endothelial Cells

**DOI:** 10.1371/journal.ppat.1000512

**Published:** 2009-07-10

**Authors:** Whitney Greene, Shou-Jiang Gao

**Affiliations:** 1 Tumor Virology Program, Greehey Children's Cancer Research Institute, The University of Texas Health Science Center at San Antonio, San Antonio, Texas, United States of America; 2 Department of Pediatrics, The University of Texas Health Science Center at San Antonio, San Antonio, Texas, United States of America; 3 Department of Microbiology and Immunology, The University of Texas Health Science Center at San Antonio, San Antonio, Texas, United States of America; 4 Cancer Therapy and Research Center, The University of Texas Health Science Center at San Antonio, San Antonio, Texas, United States of America; 5 Tumor Virology Group, Wuhan Institute of Virology, Chinese Academy of Sciences, Wuhan, China; University of Southern California School of Medicine, United States of America

## Abstract

The role of actin dynamics in clathrin-mediated endocytosis in mammalian cells is unclear. In this study, we define the role of actin cytoskeleton in Kaposi's sarcoma-associated herpesvirus (KSHV) entry and trafficking in endothelial cells using an immunofluorescence-based assay to visualize viral capsids and the associated cellular components. In contrast to infectivity or reporter assays, this method does not rely on the expression of any viral and reporter genes, but instead directly tracks the accumulation of individual viral particles at the nuclear membrane as an indicator of successful viral entry and trafficking in cells. Inhibitors of endosomal acidification reduced both the percentage of nuclei with viral particles and the total number of viral particles docking at the perinuclear region, indicating endocytosis, rather than plasma membrane fusion, as the primary route for KSHV entry into endothelial cells. Accordingly, a viral envelope protein was only detected on internalized KSHV particles at the early but not late stage of infection. Inhibitors of clathrin- but not caveolae/lipid raft-mediated endocytosis blocked KSHV entry, indicating that clathrin-mediated endocytosis is the major route of KSHV entry into endothelial cells. KSHV particles were colocalized not only with markers of early and recycling endosomes, and lysosomes, but also with actin filaments at the early time points of infection. Consistent with these observations, transferrin, which enters cells by clathrin-mediated endocytosis, was found to be associated with actin filaments together with early and recycling endosomes, and to a lesser degree, with late endosomes and lysosomes. KSHV infection induced dynamic actin cytoskeleton rearrangements. Disruption of the actin cytoskeleton and inhibition of regulators of actin nucleation such as Rho GTPases and Arp2/3 complex profoundly blocked KSHV entry and trafficking. Together, these results indicate an important role for actin dynamics in the internalization and endosomal sorting/trafficking of KSHV and clathrin-mediated endocytosis in endothelial cells.

## Author Summary

Endocytosis, an essential biological process mediating cellular internalization events, is often exploited by pathogens for their entry into target cells. The role of actin cytoskeleton in clathrin-mediated endocytosis in mammalian cells remains unclear. Kaposi's sarcoma-associated herpesvirus (KSHV) is a gammaherpesvirus linked to the development of Kaposi's sarcoma, an endothelial malignancy commonly found in AIDS patients, and several other malignancies. In this study, we found that KSHV uses the clathrin-mediated endocytosis pathway to enter endothelial cells, and this process is regulated by actin dynamics. We found KSHV particles in early and recycling endosomes, and lysosomes, which are docked on actin filaments at the early time points of viral infection. Similarly, transferrin, which enters cells by clathrin-mediated endocytosis, is associated with actin filaments together with early and recycling endosomes, and, to a lesser degree, with late endosomes and lysosomes. Disruption of the actin cytoskeleton and inhibition of regulators of actin nucleation such as Rho GTPases and Arp2/3 complex profoundly blocked KSHV entry and trafficking in endothelial cells. Together, these results define an important role for actin dynamics in multiple endosomal steps during KSHV infection and clathrin-mediated endocytosis in endothelial cells.

## Introduction

Endocytosis is a constitutive cellular process that results in the internalization of cell surface receptors and ligands, and membrane components, often initiating the activation of signal transduction cascades [1]. The endocytic pathway is often exploited by a variety of pathogens to gain entry into the cells [2].

The best-described endocytic pathway is clathrin-mediated endocytosis [3]. In this process, the clathrin-coated pits assemble at the plasma membrane and acquire cargo. The plasma membrane proceeds to invaginate and constrict to generate a clathrin-coated vesicle, which is subsequently transported to the interior of the cell, where it loses its clathrin coat and fuses with the early endosome [3]. The orderly transport of endocytic cargo from the cell exterior to the interior is highly regulated, and requires the participation of numerous lipid components and accessory proteins, as well as alterations of fine cellular structures and controlled mechanical force to overcome the physical resistance and propel the vesicle into the cell [4].

The actin cytoskeleton has been proposed to participate in either a structural role in clathrin-mediated endocytosis, or by providing the mechanical force necessary to complete endocytosis [5],[6]. The evidence for a role of actin in this process primarily comes from studies of yeast, in which actin dynamic assembly and disassembly are essential for endocytosis [7]–[9]. However, the role of actin in endocytosis in mammalian cells is less clear [5], [10]–[12]. Studies have shown a close association between components of the endocytic machinery and actin cytoskeleton [13] while regulators of actin polymerization such as Arp2/3 and neural Wiskott-Aldrich syndrome protein (N-WASP) are found to be recruited to clathrin-coated vesicles during endocytosis [14]. However, chemical disruption of actin dynamics has resulted in only partial inhibition of endocytosis in mammalian cells [10]–[12],[15]. Since these studies analyzed endocytosis in the entire population of cells, it is possible that the results may have been confounded by the use of an alternate non-clathrin-dependent pathway, or the requirement for actin in only specific subsets of clathrin-coated vesicles [16]. In addition, since mammalian cells use actin to maintain plasma membrane tension, reduced plasma membrane tension caused by actin disruption may actually enhance invaginations during the initiation of endocytosis [17]–[19]. Endocytosis occurring at the apical surface of MDCK cells seems to be actin-dependent, while basolateral endocytosis does not appear to require an intact actin network [15]. Internalization of influenza virus by epithelial cells requires an intact actin cytoskeleton at the apical surface, while viral entry at the basolateral surface did not [20]. More recent studies using total internal reflection fluorescence microscopy (TIR-FM) technology to observe individual endocytic events recorded a burst of actin polymerization with concomitant internalization of clathrin-coated pits and movement of clathrin-coated vesicles into the cell interior, but did not determine the potential effects of actin disruption on these events [16],[21],[22].

The subcortical actin network that lies immediately beneath the plasma membrane is tethered to the membrane via linking molecules such as ezrin [23] and cortactin [24], and may participate in endocytosis by providing the mechanical force to deform the plasma membrane, allowing invagination and scission of clathrin-coated pits and/or caveolae pits [5]. Actin may also participate in later steps of the endocytic pathway by facilitating the movement of endosomes towards the cell interior [5],[16]. However, the exact role of the actin cytoskeleton in endosomal sorting and trafficking remains largely unknown.

The dynamic nature of the actin cytoskeleton is essential for its function; existing actin filaments undergo severing and depolymerization in response to cellular requirements and stimuli [25],[26] while new actin filaments are polymerized from monomeric actin subunits and by branching off from existing filaments [27]. These processes are regulated by Rho GTPases, WASP/N-WASP/WAVE, the ARP2/3 complex, and the formin family of actin-binding proteins [27]–[29].

Studies of clathrin-mediated endocytosis typically utilize fluorescently labeled molecules such as transferrin to assess the role of various accessory and regulatory proteins in endocytosis. Although transferrin and its receptor are well-known to be internalized via clathrin-mediated endocytosis, they primarily enter the recycling endosomal pathway [30]–[34], and thus, may not be entirely relevant to molecules that progress to other endosomal pathways, such as the lysosomal pathway. Several microbial pathogens exploit the endocytic pathway to gain access into the cell interior [2], and some such as *Listeria monocytogenes* are also known to specifically manipulate the actin cytoskeleton to travel through the cell [35]–[37]. However, viruses, which are inert particles that rely on existing cellular pathways to enter the target cell, make ideal candidates to study the endocytic processes.

Enveloped viruses such as Herpesviruses enter cells either by direct fusion between viral envelope and the plasma membrane or by pH-dependent fusion between the viral envelope with the endosomal membrane, followed by trafficking to the nucleus to initiate infection [38],[39]. The route of viral entry can differ between cell types [40]–[42]. Kaposi's sarcoma-associated herpesvirus (KSHV) is a γ2-herpesvirus implicated as the causative agent of Kaposi's sarcoma (KS) and primary effusion lymphoma (PEL). KS lesions are primarily composed of KSHV-infected spindle cells with endothelial markers, suggesting that the *in vivo* targets of KSHV might be endothelial cells [43]. Previous studies have shown that KSHV infects fibroblasts via clathrin-mediated endocytosis, and that microtubules are not required for virus internalization but required for the trafficking of viral particles [44],[45]. In addition, KSHV entry induces RhoA GTPase, and rearrangements of both microtubules and the actin cytoskeleton in fibroblasts [46], and RhoA GTPase is important for virus entry in HEK293 cells [47] and association of viral particles with microtubules in dermal microvascular cells (DMVEC) [48]. Nevertheless, in these studies the actin cytoskeleton was found to be involved in neither virus internalization nor virus trafficking though others have shown a potential role for the actin cytoskeleton in clathrin-mediated endocytosis [5],[13],[14],[16]. It is possible that the requirement for actin dynamics during endocytosis may differ considerably among different cell types. In addition, KSHV entry and trafficking, as well as the role of actin cytoskeleton in clathrin-mediated endocytosis in endothelial cells, have not been extensively examined so far.

To better understand the potential role of the actin cytoskeleton in KSHV infection in endothelial cells, we have used an immunofluorescence-based assay (IFA) to visualize viral capsids during KSHV entry and trafficking in the target cells. This assay does not rely on the expression of any viral genes, but instead directly visualizes the accumulation of viral capsids at the nuclear membrane as an indicator of successful viral entry and trafficking. To distinguish between plasma membrane fusion and endocytosis, cells were treated with inhibitors of endosomal acidification before and during infection. In addition, cells were examined for the presence of enveloped viral particles at the early stage of KSHV infection. The results suggested endocytosis as the primary route of entry used by KSHV to infect primary human umbilical vein endothelial cells (HUVEC). Additional studies indicated that clathrin-mediated endocytosis is the major route of KSHV entry into endothelial cells, and KSHV infection induces dynamic rearrangement of actin cytoskeleton. Both KSHV viral particles and transferrin were observed to colocalize with actin filaments and markers of endosomal maturation. Disruption of the actin cytoskeleton and inhibition of regulators of actin nucleation such as Rho GTPases and Arp2/3 complex with chemical agents profoundly reduced viral entry and trafficking, suggesting that actin dynamics might play an important role in multiple endosomal steps during KSHV infection and clathrin-mediated endocytosis in endothelial cells.

## Results

### Visualization of entry into cells and trafficking to the perinuclear regions of KSHV particles

**Figure 1:**
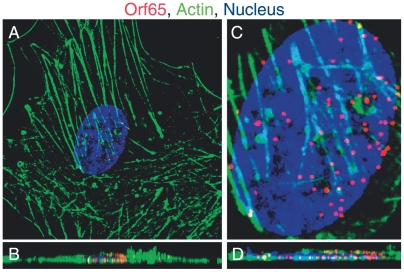
Accumulation of Orf65+ viral particles at the nucleus of a KSHV-infected endothelial cell. HUVEC were infected with KSHV and stained for Orf65+ viral capsids (red), actin cytoskeleton (green), and nuclei (blue) at 4 hpi. (A–B) An overview of a HUVEC infected by KSHV shown in a XY section (A, also see Video S1 for supporting information) and the corresponding YZ cross-section (B, also see Video S2 for supporting information). (C–D) Magnified 3D-projection image of the cell nucleus (C, also see a 3-D projection image in Video S3) and the corresponding cross-section of nucleus (D, also see Video S4 for supporting information).

To study the mechanism used by KSHV to infect endothelial cells, an assay to analyze the entry and trafficking of individual viral particles was developed. KSHV is an enveloped virus, with a cell-derived membrane enclosing a tegument layer and a central capsid containing the viral genome [43]. For herpesviruses that enter the cells via fusion of the viral envelope at the plasma membrane, the viral envelope glycoproteins are undesirable for monitoring virus entry and trafficking [38],[39]. The fate of the poorly-characterized tegument during KSHV entry and trafficking is also unclear [43]. Thus, the component of the virion best suited for visualizing the entry of a viral particle into cells and subsequent intracellular trafficking is the viral capsid. The viral capsid remains intact throughout the infection process and carries the viral genome to the host nucleus. We used a monoclonal antibody directed against the KSHV small capsid protein Orf65 to stain the viral capsid in an IFA. HUVEC inoculated with KSHV were processed for IFA at various time points post-infection to visualize the Orf65+ capsids, actin filaments, and the cell nucleus. Z-stacks were acquired with a confocal laser scanning microscope and used to generate 3D projection XY overview images (Figure 1A and Video S1) and the corresponding cross-sectional YZ images (Figure 1B and Video S2). Images were further magnified to show the nucleus and individual viral particles (Figure 1C and D, and Videos S3 and S4). Within 4 h post-infection (hpi), we observed a large number of viral capsids in the range of 10–70 particles per cell at the perinuclear region. We quantified the total number of viral particles reaching the perinuclear region as an indication of successful viral entry and trafficking.

The entry of enveloped viruses such as herpesviruses can occur by two different routes: fusion of the viral envelope with the plasma membrane followed by delivery of the viral particles into the host cytoplasm, or by attachment to a cellular receptor(s) and subsequent endocytosis of the receptor proteins and viral particles [39]. The plasma membrane fusion route is pH-independent, while viral entry via endocytosis is pH-dependent, and most of enveloped viruses require low pH in the endosomal vesicle to initiate viral envelope fusion with the endosomal membrane. In both cases, the viral capsids are required to traffic to the perinuclear regions and deliver the viral genome into the nucleus to complete the infection process. We co-stained the KSHV-infected cells for both Orf65+ capsids and glycoprotein B (gB), a viral envelope protein. The cells were visualized with an epifluorescence microscope. If a virion enters the cells via plasma membrane fusion, it should lose its envelope at the time of internalization or membrane fusion. However, if a virion enters the cells via endocytosis, it should retain its envelope immediately after internalization but lose it when subsequent fusion with endosomal membrane occurs. At 5 min post-infection (mpi), over 90% of the Orf65+ particles inside the cells were positive for gB (Figure S1A). In contrast, at 4 hpi, almost all the Orf65+ particles inside the cells were negative for gB (Figure S1B). As expected, at both time points, 100% of the Orf65+ particles outside the cells were positive for gB (Figure S1A and B). These results suggest that KSHV likely enters endothelial cells by endocytosis rather than plasma membrane fusion.

### Inhibition of endosomal acidification prevents entry and trafficking of KSHV particles to nucleus

**Figure 2:**
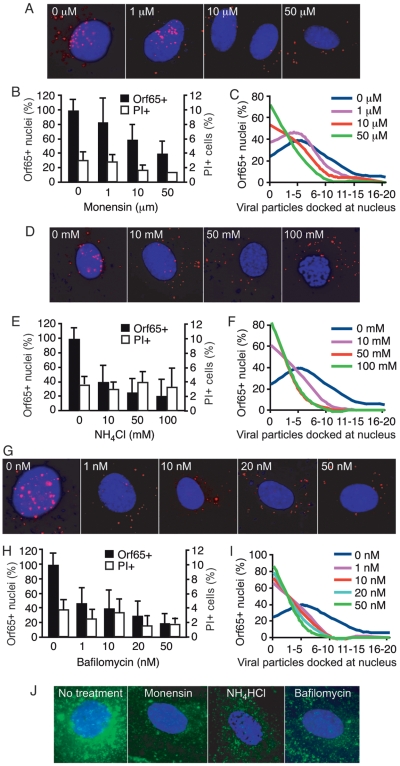
Inhibition of endosomal acidification reduces the entry and trafficking of KSHV particles to the perinuclear region. (A, D, and G) HUVEC were treated with chemical inhibitors of endosomal acidification at indicated doses for 1 h prior to inoculation with KSHV. Cells were inoculated with KSHV in the presence of inhibitors, fixed at 8 hpi, and stained for Orf65+ viral particles (red) and nuclei (blue). (B, E and H) The total number of nuclei bearing at least one Orf65+ particle was determined and calculated as Orf65+ nuclei (%). In parallel experiments, cells were subjected to the same treatments and evaluated for viability by PI staining. (C, F and I) The total number of Orf65+ particles docked at each nucleus was determined. (J) Inhibition of endosomal acidification reduces the internalization of AlexaFluor 488-transferrin. HUVEC were pretreated with inhibitors of clathrin assembly for 1 h prior to exposure to AlexaFluor 488-transferrin. The reduced internalization and perinuclear accumulation of transferrin (green) compared to untreated control cells demonstrates the effective inhibition of endosomal acidification.

To further analyze the role of endocytosis in KSHV entry into endothelial cells, we used a number of chemical inhibitors of endosomal acidification. NH_4_Cl is an acidotropic weak-base amine used to nonspecifically increase intravesicular pH [49]. Monensin is a sodium ionophore that is able to cross membranes and bind monovalent cations, resulting in an increase in endosomal pH [50]. Bafilomycin A1 is a potent inhibitor of the vacuolar ATPase and specifically prevents acidification of endosomal vesicles [51]. HUVEC pretreated with inhibitors for 1 h were inoculated with KSHV in the presence of the inhibitors, fixed at 8 hpi, and stained for Orf65+ capsids and nuclei. The cells were then visualized with an epifluorescence microscope, and the percentage of the nuclei with at least one Orf65+ particle as well as the total number of viral particles per nucleus was counted (Figure 2). While other methods such as infectivity and reporter assays have been used for monitoring the effects of inhibitors on viral entry and trafficking, they often rely on the expression of viral and reporter genes, and thus, can not distinguish the entry events of single versus multiple viral particles. In contrast, this method directly measures the entry and trafficking of individual viral particles to the perinuclear region. As shown in Figure 2A, B, D, E, G, and H, all inhibitors of endosomal acidification tested significantly reduced the total number of nuclei bearing at least one Orf65+ viral particle in a dose-dependent manner, suggesting that endocytosis rather than plasma membrane fusion, is the primary route of entry into HUVEC used by KSHV. These results are consistent with a previous study using viral infectivity as a method to monitor KSHV entry of human fibroblasts [44]. The effect of inhibition of endosomal acidification becomes more apparent when the absolute numbers of viral particles successfully reaching the nucleus is quantified (Figure 2C, F and I). The nuclei of untreated cells had significantly more Orf65+ viral particles than the nuclei from cells treated with inhibitors. To ensure that the observed results were not due to the side effects of the inhibitors, we examined the viability of the cells. HUVEC were subjected to the same treatments with inhibitors and stained with propidium iodide (PI). None of the inhibitors increased the number of PI+ cells even at the highest concentrations used (Figure 2B, E and H, and Figure S2). These chemical inhibitors also efficiently inhibited the endocytic trafficking of AlexaFluor 488-transferrin, thus demonstrating their effectiveness in inhibiting endosomal maturation (Figure 2J). Together, these results suggest that KSHV requires endosomal acidification to efficiently infect endothelial cells and traffic to the perinuclear region.

### Inhibition of clathrin-coated pits assembly prevents entry and trafficking of viral particles to the nucleus

**Figure 3:**
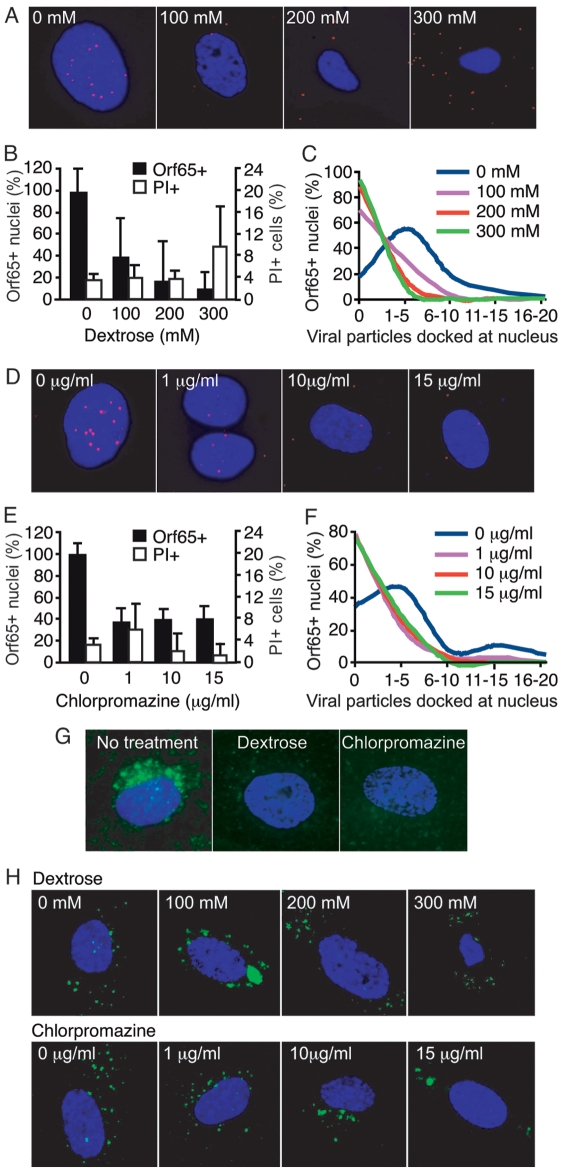
Inhibition of clathrin assembly reduces the entry and trafficking of KSHV particles to the perinuclear region. (A and D) HUVEC were treated with chemical inhibitors of clathrin assembly at the indicated doses for 1 h prior to inoculation with KSHV. Cells were inoculated with KSHV in the presence of inhibitors, fixed at 8 hpi (dextrose) or 2 hpi (chlorpromazine), and stained for Orf65+ viral particles (red) and nuclei (blue). (B and E) The total number of nuclei bearing at least one Orf65+ particle was determined and calculated as Orf65+ nuclei (%). In parallel experiments, cells were subjected to the same treatments and evaluated for viability by PI staining. (C and F) The total number of Orf65+ particles docked at each nucleus was determined. (G–H) Inhibition of clathrin assembly reduces the internalization of AlexaFluor 488-transferrin (G) but not AlexaFluor 488-CTB (H). HUVEC were pretreated with inhibitors of clathrin assembly for 1 h prior to exposure to AlexaFluor 488-transferrin or AlexaFluor 488-CTB. The reduced internalization and perinuclear accumulation of transferrin (green) compared to untreated control cells demonstrates the effective inhibition of clathrin-mediated endocytosis while the internalization and perinuclear accumulation of CTB (green) indicates that the inhibitors did not affect caveolae-mediated endocytosis.

**Figure 4:**
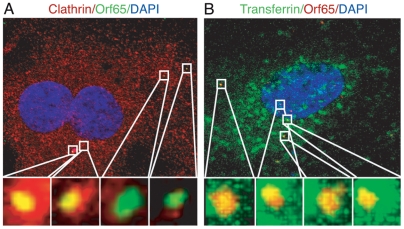
Colocalization of Orf65+ KSHV particles with markers of clathrin-mediated endocytosis during KSHV infection of endothelial cells. (A) HUVEC were inoculated with KSHV, fixed at 1 hpi and stained for clathrin heavy chain (red) and Orf65+ viral particles (green). (B) HUVEC were infected with KSHV and simultaneously labeled with Alexafluor 488-transferrin (green). Cells were fixed at 1 hpi and stained for Orf65+ viral particles (red). Regions highlighted in the squares in the upper images are shown as enlarged 3D-projection images in the lower panels. Areas of colocalization of red and green signals are revealed as yellow. For corresponding 3D projection of colocalization of Orf65+ particles with clathrin, see Videos S5, S6, S7 and S8. For corresponding 3D projection of colocalization of Orf65+ particles with transferrin, see Videos S9, S10, S11 and S12.

Mammalian cells utilize multiple endocytic pathways, including macropinocytosis, clathrin-mediated endocytosis, caveolae-mediated endocytosis, and a poorly understood non-clathrin, non-caveolae-mediated endocytosis pathway [4]. During clathrin-mediated endocytosis, clathrin-coated pits assemble on the cytoplasmic side of the plasma membrane in response to internalization signals from the receptor [52]. Hypertonic media has been shown to inhibit the formation of clathrin-coated pits at the plasma membrane, while the cationic amphiphilic agent chlorpromazine causes misassembly of clathrin-coated pits and inhibits clathrin-mediated endocytosis [53]. The role of the clathrin-mediated endocytosis pathway during KSHV entry into HUVEC was analyzed by adding dextrose at 100 to 300 mM to the media to generate hypertonic conditions, or by treating the cells with chlorpromazine at 1 to 15 µg/ml. Because of the high toxicity, cells treated with chlorpromazine were fixed and stained for Orf65+ capsids at 2 hpi. Under these conditions, both inhibitors had minimal toxicity (Figure 3B and E, and Figure S3). As shown in Figure 3A, B, D and E, inhibition of clathrin assembly by either dextrose or chlorpromazine significantly reduced the total number of nuclei bearing at least one Orf65+ viral particle. In addition, inhibition of clathrin assembly also reduced the absolute number of viral particles reaching each nucleus (Figure 3C and F). The inhibitors also efficiently inhibited the internalization of AlexaFluor 488-transferrin, which is internalized by cells through clathrin-mediated endocytosis (Figure 3G); however, they did not inhibit the internalization of Cholera Toxin B (CTB), which is internalized by cells through caveolae-mediated endocytosis (Figure 3H). CTB binds GM1 ganglioside and triggers its internalization by caveolae/lipid raft-mediated endocytosis [54]–[56]. Thus, internalization of AlexaFluor 488-CTB was used as an indicator for caveolae/lipid raft-mediated endocytosis. As expected, inhibitors of caveolae-mediated endocytosis prevented the internalization of CTB (see below). These results indicated that dextrose and chlorpromazine specifically inhibited clathrin- but not caveolae-mediated endocytosis. Furthermore, in a separate experiment, we found colocalization of KSHV capsids with clathrin (Figure 4A, and Videos S5, S6, S7 and S8) and AlexaFluor 488-transferrin (Figure 4B, and Videos S9, S10, S11 and S12). Together, these results suggest that KSHV entry of endothelial cells is primarily mediated by clathrin-mediated endocytosis.

### Inhibition of caveolae/lipid raft-mediated endocytosis does not prevent the entry and trafficking of KSHV particles

**Figure 5:**
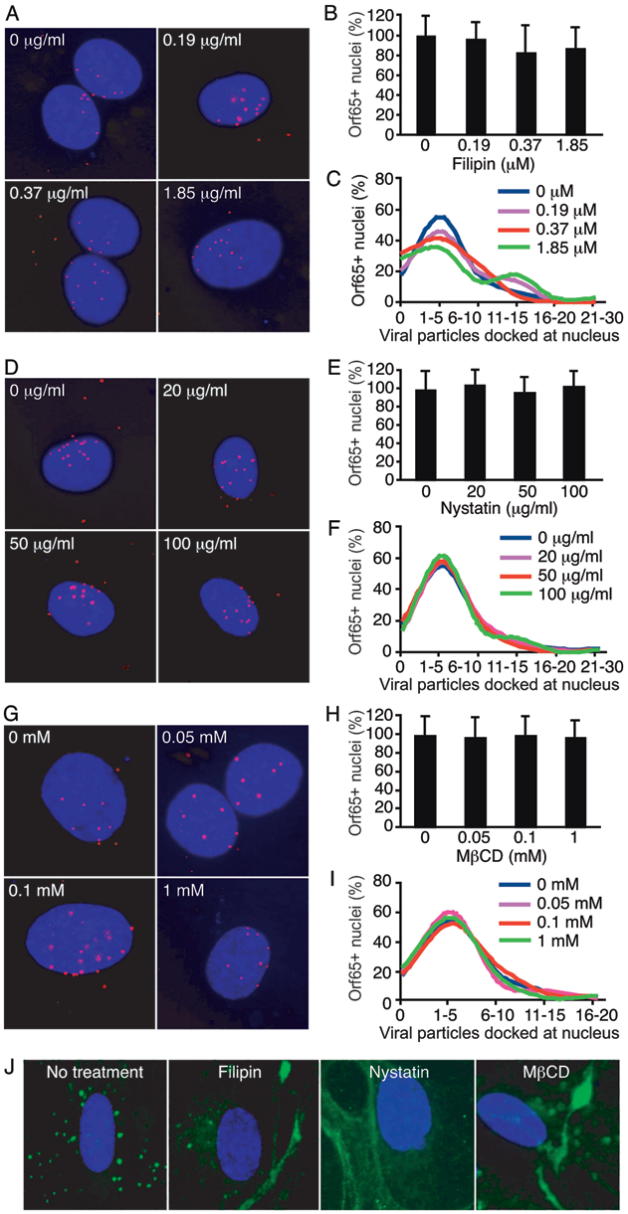
Inhibition of caveolae/lipid raft-mediated endocytosis fails to reduce the entry and trafficking of KSHV particles to the perinuclear region. (A, D and G) HUVEC were treated with chemicals to deplete membrane cholesterol and inhibit caveolae/lipid raft-mediated endocytosis at the indicated doses for 1 h. Cells were inoculated with KSHV in the presence of inhibitors, fixed at 8 hpi, and stained for Orf65+ viral particles (red) and nuclei (blue). (B, E and H) The total number of nuclei bearing at least one Orf65+ viral particle was determined. (C, F and I) The total number of Orf65+ viral particles docked at each nucleus was determined. (J) Inhibition of caveolae/lipid raft-mediated endocytosis reduces the internalization of AlexaFluor 488-CTB. HUVEC were pretreated with inhibitors of caveolae/lipid raft-mediated endocytosis for 1 h prior to exposure to AlexaFluor 488-CTB. The reduced internalization and increased membrane accumulation of CTB (green) compared to untreated control cells demonstrates the effective inhibition of caveolae/lipid raft-mediated endocytosis.

Several viruses such as HIV-1, SV40, Ebola virus, Marburg virus, polyoma virus and echovirus use caveolae/lipid raft-mediated endocytosis to infect cells [57]. EBV entry into lymphocytes also requires cholesterol [58]. Endothelial cell membranes are highly enriched in caveolae, and uptake of albumin for transcytosis by these cells is facilitated by caveolae-mediated endocytosis [59],[60], suggesting that caveolae-mediated endocytosis in endothelial cells is a highly efficient process that could be exploited by viruses for entry into cells. We examined the involvement of caveolae-mediated endocytosis in KSHV entry into HUVEC. The chemical agents filipin, nystatin, and methyl-β-cyclodextrin (MβCD) deplete cholesterol from the plasma membrane and prevent caveolae/lipid raft-mediated endocytosis [57]. These agents were used at different concentrations to determine the role of caveolae/lipid rafts in KSHV entry. As shown in Figure 5, none of these inhibitors had any effect on KSHV entry into HUVEC. The percentage of nuclei in the drug-treated cells bearing at least one Orf65+ viral particle was similar to the untreated controls (Figure 5A, B, D, E, G, and H). Similarly, the absolute number of viral particles attached to each nucleus was not affected by any of the inhibitors (Figure 5C, F and I). As controls, the inhibitors of caveolae/lipid raft-mediated endocytosis effectively prevented the internalization of CTB resulting in membrane accumulation of the marker (Figure 5J). These results indicate that KSHV was not prevented from entering the cells and trafficking to the perinuclear region though the chemicals successfully inhibited caveolae/lipid raft-mediated endocytosis. These results are consistent with a previous study showing that nystatin and MβCD do not have any effect on KSHV entry into fibroblasts [44]. However, they contradict a later report showing that MβCD affects the association with microtubules and intracellular trafficking of viral particles in DMVEC [48]. Thus, there might be an alternative route for trafficking of KSHV particles in HUVEC.

### KSHV induces dynamic remodeling of the actin cytoskeleton during entry into endothelial cells

**Figure 6:**
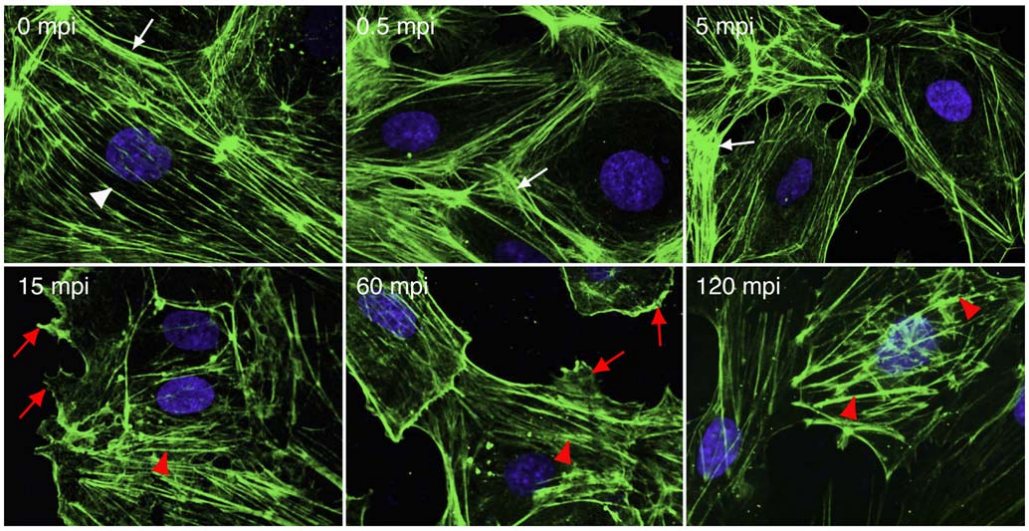
KSHV infection of endothelial cells induces actin dynamics and rearrangements of the actin cytoskeleton. HUVEC were infected with KSHV and stained for actin cytoskeleton (green) and nuclei (blue) at different time points post-infection. Actin stress fibers (while arrow head) and cortical actin structures (white arrow) were visible before viral infection. KSHV infection disrupted actin stress fibers and induced more cortical actin structures at the early stage of infection (<15 mpi). However, cortical actin structures started to dissolve accompanying the reappearance of short and thick actin filaments resembling actin tails/spikes (red arrow head) after 15 mpi. Membrane ruffling, lamellipodia and filopodia were visible after 15 mpi (red arrow head).

While the actin cytoskeleton was not involved in KSHV entry and trafficking in fibroblasts [44], recombinant KSHV gB was sufficient to induce the protrusion of lamellipodia and filopodia [46], both of which depend on actin polymerization [5]. We examined the rearrangements of the actin cytoskeleton during KSHV infection (Figure 6). Before infection, HUVEC had distinct actin stress fibers (white arrow head) and cortical actin structures (white arrow). At as early as 0.5 mpi, we observed the dissolution of the actin stress fibers and increased intensity of the cortical actin structures, which continued for over 10 min. However, at 15 mpi, we started to observe the growth of new actin filaments, and dissolution of the cortical actin structures. At the same time, we also observed membrane ruffling, lamellipodia and filopodia (red arrow). The new actin filaments were shorter and thicker than the stress fibers observed before viral infection, and were sustained for up to 120 mpi, at which time point, they resembled distinct actin tails or spikes (red arrow head). These results indicate that KSHV induces dynamic remodeling of the actin cytoskeleton at the early stages of infection in endothelial cells.

### Chemical disruption of actin cytoskeleton inhibits KSHV entry and trafficking in endothelial cells

**Figure 7:**
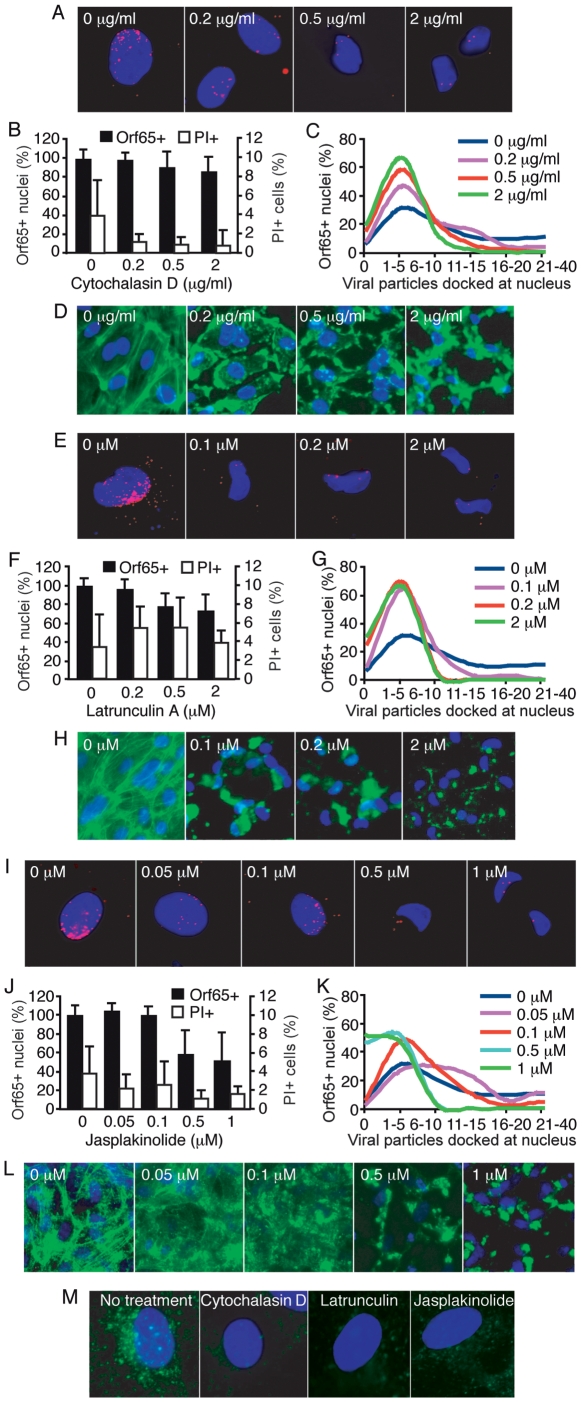
Disruption of actin dynamics reduces the entry and trafficking of KSHV particles to the perinuclear region. (A, E and I) HUVEC were treated with chemicals to disrupt actin dynamics at the indicated doses for 1 h. Cells were inoculated with KSHV in the presence of inhibitors, fixed at 4 hpi, and stained for Orf65+ viral particles (red) and nuclei (blue). (B, F and J) The total number of nuclei bearing at least one Orf65+ viral particle was determined and calculated as Orf65+ nuclei (%). In parallel experiments, cells were subjected to the same treatments and evaluated for viability by PI staining. (C, G and K) The total number of Orf65+ viral particles docked at each nucleus was determined. (D, H and L) Disruption of the actin cytoskeleton by cytochalasin (D), latrunculin A (H) and jasplakinolide (L). HUVEC were treated with the inhibitors as described in A, E and I, and stained for the actin cytoskeleton with AlexaFluor 488-phalloidin except for cells treated with jasplakinolide, which were stained with an antibody to β-actin. (M) Disruption of actin dynamics reduces the internalization of AlexaFluor 488-transferrin. HUVEC were pretreated with chemicals to disrupt actin dynamics for 1 h prior to exposure to AlexaFluor 488-transferrin. The reduced internalization and perinuclear accumulation of transferrin (green) compared to untreated control cells demonstrates the effective inhibition of clathrin-mediated endocytosis when actin dynamics are disrupted.

We further examined the role of actin cytoskeleton in KSHV entry and trafficking in endothelial cells by using chemicals to disrupt their dynamics. Latrunculin A reversibly disrupts actin dynamics by targeting monomeric G-actin and preventing actin polymerization [61],[62]. Cytochalasin D reversibly targets F-actin, inducing depolymerization of existing actin filaments and increasing the cellular pool of ADP-bound actin monomers [63],[64]. Jasplakinolide reversibly inhibits normal cellular actin dynamics by hyperstabilizing actin filaments, preventing depolymerization, and depleting the cellular pool of free actin monomers available for *de novo* polymerization [65]. Disruption of actin dynamics did not significantly reduce the total number of nuclei bearing at least one Orf65+ viral particle (Figure 7B, F and J). Although these results are consistent with previous observations that the actin cytoskeleton was not involved in KSHV entry and trafficking in fibroblasts measured by non-viral particle-based assays [44], when the total number of viral particles docked at each nucleus was quantified, the cells treated with the actin-disrupting agents had significantly fewer viral particles per nucleus than those of untreated cells (Figure 7A, C, E, G, I and K). In addition, the effect of inhibition also varied with the actin-disrupting agents, which could reflect their distinct modes of action. These results suggest that actin dynamics are essential for the entry of most KSHV particles; however, it appears that a small number of viral particles are able to enter the cells even in the absence of a functional actin cytoskeleton system, which could partially account for the failure to observe an effect of actin-disrupting agents on KSHV entry and trafficking when measured with non-viral particle-based infectivity assays, especially when high viral multiplicity of infection (MOI) was used [44]. Under our experimental conditions, we did not observe any effect of these inhibitors on cell viability (Figure 7B, F and J, and Figure S4). Examination of the actin cytoskeleton showed that they were effectively disrupted by all three inhibitors (Figure 7D, H and L). Internalization of Alexafluor 488-transferrin and accumulation in the perinuclear region was used as an indicator of active clathrin-mediated endocytosis. As shown in Figure 7M, internalization of transferrin was inhibited by the disruption of actin dynamics, resulting in reduced levels of transferrin in the perinuclear region of the treated cells, further illustrating the important role of actin dynamics during endocytosis in endothelial cells.

**Figure 8:**
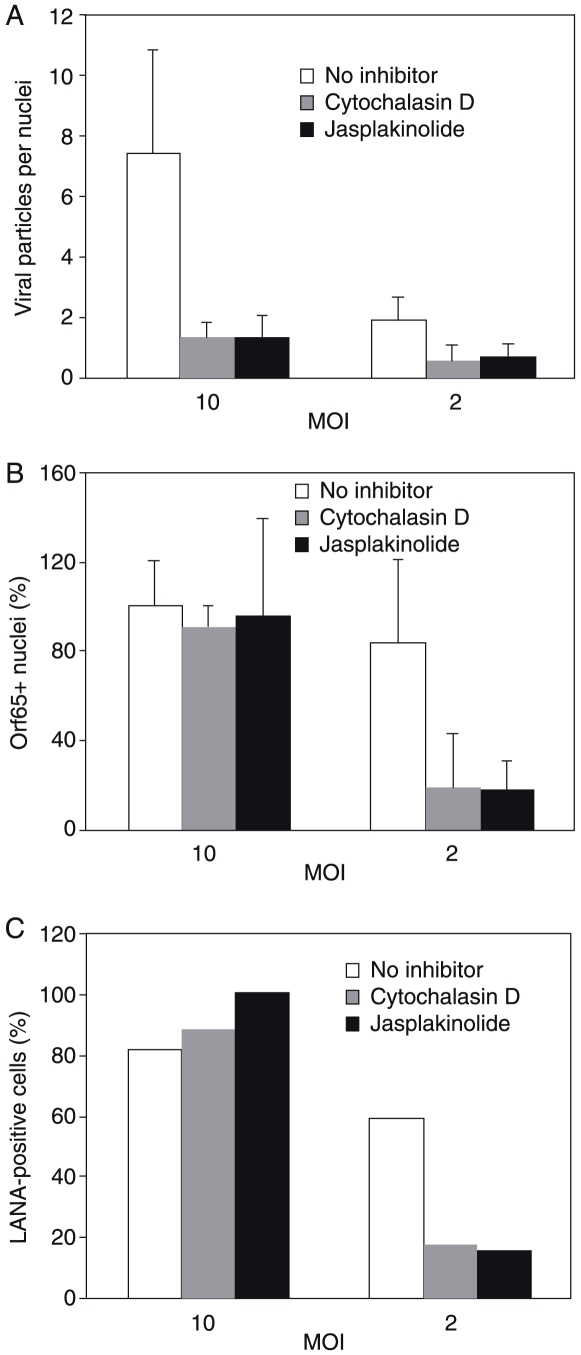
Disruption of actin dynamics reduces KSHV infectivity at low MOI but not high MOI. HUVEC were infected with KSHV at a high MOI (10) and a low MOI (2). The effects of actin cytoskeleton-disrupting agents cytochalasin D at 2 µg/ml and jasplakinolide at 1 µM on the average number of viral particles per nuclei (A), and viral infectivity based on the percentage of nuclei containing at least one viral particle (B) or the percentage of LANA-positive cells (C) were examined.

While disruption of actin dynamics affected the entry of KSHV particles, our results indicated that its effect on KSHV infectivity might only be obvious at a low MOI. To confirm these observations, we infected HUVEC at 10 and 2 MOI. Since there is no plaque assay for KSHV, we quantified viral MOI based on the number of internalized viral particles per cell. To track viral infectivity, we stained the cells for LANA and counted the percentage of LANA-positive cells. As expected, cytochalasin D and jasplakinolide effectively reduced the average numbers of viral particles per nuclei (Figure 8A). At a high MOI (10), these inhibitors did not have any effects on the percentage of cells containing at least one viral particle (Figure 8B), as well as viral infectivity measured by the percentage of LANA-positive cells (Figure 8C). In contrast, at a low MOI, both cytochalasin D and jasplakinolide effectively reduced the percentage of cells containing at least one viral particle (Figure 8B) and viral infectivity (Figure 8C). Thus, the effect of disrupting actin dynamics on KSHV infectivity is MOI-dependent.

### Chemical inhibition of regulators of actin nucleation prevents the entry and trafficking of KSHV particles to the nucleus

**Figure 9:**
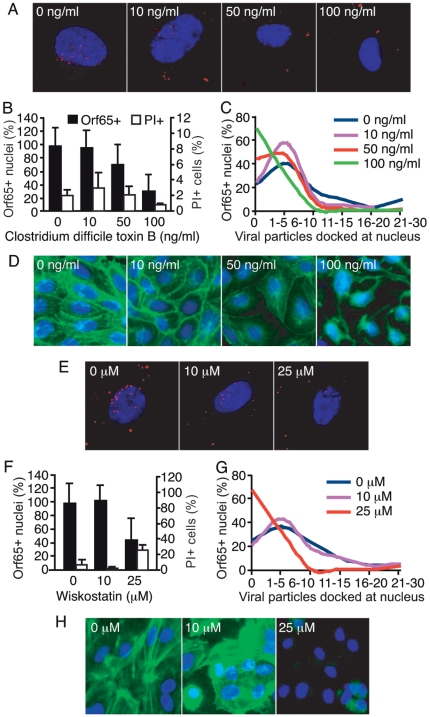
Disruption of actin regulators reduces the entry and trafficking of KSHV particles to the perinuclear region. (A and E) HUVEC were treated with chemicals to inhibit Rho GTPase function (A) or Arp2/3 complex activity (E) at the indicated doses for 1 h prior to inoculation with KSHV. Cells were inoculated with KSHV in the presence of inhibitors, fixed at 4 hpi, and stained for Orf65+ viral particles (red) and nuclei (blue). (B and F) The total number of nuclei bearing at least one Orf65+ particle was determined. In parallel experiments, cells were subjected to the same treatments and evaluated for viability by PI staining. (C and G) The total number of Orf65+ particles docked at each nucleus was determined. (D and H) Disruption of the actin cytoskeleton by CdTB (D) and wiskostatin (H). HUVEC were treated with the inhibitors as described in A and E, and stained for the actin cytoskeleton with AlexaFluor 488-phalloidin.

The Rho family of small GTPases such as Rho, Rac and Cdc42 are important regulators of actin cytoskeletal assembly and organization, as well as intracellular trafficking events during endocytosis [66]. Rho induces the formation of stress fibers, while activation of Rac and Cdc42 induces the polymerization of actin and the formation of a network of actin filaments underlying the plasma membrane [67]. Considering these known functions, Rho GTPases are excellent candidates for mediating the signaling between actin and endocytic traffic [68], and may therefore be involved in the endocytic entry and trafficking of KSHV. *Clostridium difficile* Toxin B (CdTB) glucosylates and inactivates Rho GTPases, leading to the disruption of actin dynamics [69]. A previous study showed that KSHV infection of HEK293 cells activated RhoA GTPase and treatment with CdTB inhibited the internalization of KSHV DNA in fibroblasts and DMVEC [47]. However, whether these effects were mediated by actin dynamics and whether the same effects are also present in HUVEC remain unclear. To examine the role of Rho GTPases in KSHV entry of HUVEC, cells were treated with CdTB at different concentrations before and during infection with KSHV. CdTB significantly reduced the total number of nuclei bearing at least one Orf65+ viral particle (Figure 9A and B). The absolute number of Orf65+ viral particles per nucleus was also reduced in a dose-dependent fashion (Figure 9A and C). As expected, treatment with CdTB led to the disruption of the actin cytoskeleton (Figure 9D); however, it did not affect the viability of the cells (Figure 9B and Figure S5A). These results implicate a role for Rho GTPases in KSHV entry and trafficking to the nucleus.

Following the activation of Rho GTPases, actin filament assembly is regulated by N-WASP and the Arp2/3 complex. N-WASP activity is required for the activation of Arp2/3. Both Arp2/3 and N-WASP are recruited to sites of clathrin-mediated endocytosis [27],[70]. Arp2/3 binds to existing actin filaments and directly nucleates new actin filaments to form a branched network [27]. Wiskostatin is a cell-permeable chemical that inhibits N-WASP by stabilizing the auto-inhibited conformation, and thus preventing the activation of the Arp2/3 complex [71]. To further investigate the role of *de novo* actin assembly in viral entry and trafficking, HUVEC were pretreated with wiskostatin, infected with KSHV, and stained for Orf65+ capsids. At 25 µM, wiskostatin decreased the total number of nuclei with at least one Orf65+ viral particle (Figure 9E and F). In addition, the absolute number of viral particles docked at each nucleus was reduced in wiskostatin-treated cells as compared to the untreated cells (Figure 9G). Similar to CdTB, treatment with wiskostatin also led to the disruption of the actin cytoskeleton (Figure 9H) but it had minimal effect on cell viability (Figure 9F and Figure S5B). Thus, the regulation of actin polymerization through the WASP family members and Arp2/3 activation is required for KSHV particles to enter HUVEC and traffic to the perinuclear region. These results are consistent with those of Figure 7, and Figure 9A, B, C and D, and demonstrate the importance of *de novo* actin assembly and regulation of actin dynamics during KSHV entry.

### KSHV particles are associated with actin filaments at different endosomal steps during KSHV entry and trafficking in endothelial cells

**Figure 10:**
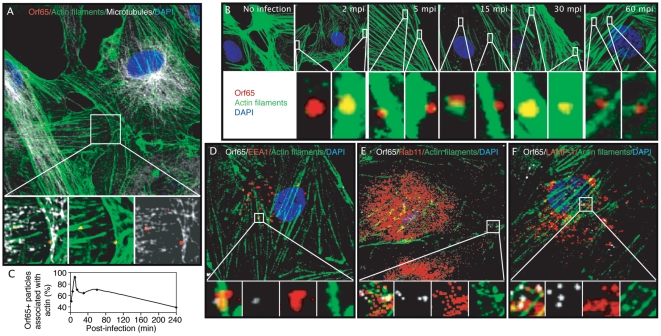
Association of Orf65+ KSHV capsids with actin filaments at different stages of endocytosis during KSHV entry and trafficking in endothelial cells. (A) HUVEC were infected with KSHV, fixed at 30 mpi, stained for Orf65+ viral capsids (red), actin filaments (green), and microtubules (white). The area highlighted by the square is enlarged and projected as 3D-projection images in the lower panels, which reveal the association of Orf65+ viral particles with actin filaments (middle), microtubules (right), and the merged image (left). (B) Time course analysis of the association Orf65+ viral particles with actin filaments. HUVEC were infected with KSHV, fixed at indicated time points post-infection, and stained for actin filaments (green), Orf65+ viral particles (red), and nuclei (blue). Highlighted sections in the upper panels were enlarged and shown as 3D-projection images in the bottom panels, which demonstrate the association of viral capsids with actin filaments at different time points of infection. (C) Quantification of the kinetics of colocalization of Orf65+ particles with actin filaments. (D–F) Association of Orf65+ viral particles shown in white with the endosomal protein EEA1 (D), Rab11 (E), or LAMP-1 (F), all in red, and actin filaments in green during KSHV entry of endothelial cells. HUVEC were prepared as described in (A) and labeled to visualize endosomal proteins, Orf65+ viral capsids and actin filaments. The sections highlighted by the squares are enlarged in the lower panels. For corresponding 3D projection of colocalization of Orf65+ particles with actin filaments and EEA1, Rab11 and LAMP-1, see Videos S13, S14 and S15.

While a previous study has shown that disruption of lipid raft with MβCD and nystatin enhanced KSHV internalization but inhibited subsequent microtubule-mediated trafficking of viral particles in DMVEC [48], our results have shown that neither inhibitor has any effect on the internalization and trafficking of viral particles but actin cytoskeleton-disrupting agents do. We speculated that actin dynamics might be involved in the endosomal sorting and/or intracellular trafficking of KSHV particles in HUVEC. It has been shown that the role of actin in endocytosis is variable in mammalian cells depending upon the cell type and/or the ligand/receptor. To further confirm the role of actin dynamics in clathrin-mediated endocytosis during KSHV entry and trafficking in endothelial cells, HUVEC were infected with KSHV for 30 min, fixed and stained for Orf65+ capsids, actin filaments, microtubules and the cell nucleus. As shown in confocal images (Figure 10A), ORF65+ viral particles were closely associated with both actin fibers and microtubules.

We next sought to determine at what point during the viral entry and trafficking process actin might be involved. In a time-course experiment, HUVEC were inoculated with KSHV and fixed at 2, 5, 15, 30, and 60 mpi. Figure 10B reveals colocalization of actin filaments with ORF65+ KSHV particles at all time points examined. The association of KSHV particles with actin filaments peaked at around 10–15 mpi reaching over 90% of all viral particles before it started to drop (Figure 10C). Interestingly, we observed the association of viral particles with actin filaments as late as 4 hpi albeit endocytosis is a relative rapid process. However, such association with actin filaments at the later time points of infection might simply reflect the unsynchronized nature of the infection. Nevertheless, these results confirmed a potential role for actin dynamics in multiple steps of clathrin-mediated endocytosis, and KSHV entry and trafficking in endothelial cells.

To further identify the stage of endocytosis during KSHV entry and trafficking in endothelial cells that might be regulated by actin dynamics, HUVEC were infected with KSHV, and stained for markers of endosomes as well as Or65+ KSHV particles and actin filaments. The image in Figure 10D reveals the colocalization of Orf65+ viral capsids, actin filaments, and EEA1, a marker for the early endosome (Video S13). Figure 10E reveals the association of viral capsids with actin filaments, and Rab11, a marker for the recycling endosome (Video S14). Figure 10F demonstrates the colocalization of viral capsids with actin filaments, and LAMP-1, a marker for the late endosome/lysosome (Video S15). These results suggest that actin dynamics are involved in multiple steps of endocytosis and endosomal trafficking of KSHV, from the early endosome through the late endosome/lysosome.

### Transferrin is associated with actin filaments at different endosomal steps during endocytosis in endothelial cells

**Figure 11:**
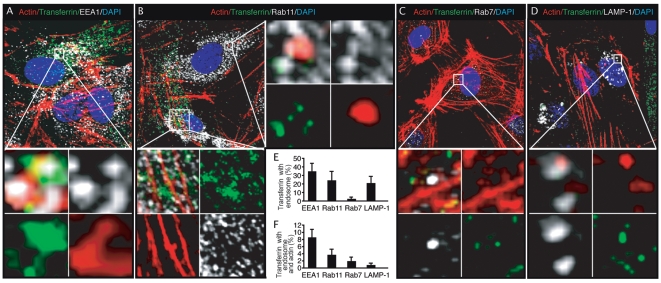
Association of Alexafluor 488-transferrin with actin filaments at different stages of endocytosis in endothelial cells. (A–D) HUVEC were treated with Alexafluor 488-transferrin for 60 min, fixed and stained for actin filaments (red), markers of endosomes (white) and nuclei (blue). Alexafluor 488-transferrin shown in green was clearly associated with actin filaments and endosomal protein EEA1 (A), Rab11 (B), Rab7 (C) and LAMP-1 (D). The sections highlighted by the squares are enlarged in the lower or right panels. For corresponding 3D projection of colocalization of Alexafluor 488-transferrin with actin filaments and EEA1, Rab11, Rab7 and LAMP-1, see Videos S16, S17, S18, S19 and S20. (E–F) Quantification of colocalization of Alexafluor 488-transferrin with markers of endosomes (E), and with both actin filaments and markers of endosomes (F).

The association of KSHV particles with both actin filaments and markers of early and recycling endosome, and lysosome suggest that actin dynamics might be involved in several post-internalization steps of endocytosis including endosome sorting and trafficking in endothelial cells. We further examined the association of Alexafluor 488-transferrin with actin filaments, and markers of endosomes (Figure 11). HUVEC were treated with Alexafluor 488-transferrin for 60 min, fixed and stained for early, intermediate, and late endosomes as well as actin filaments. Consistent with previous observations in epithelial cells that the internalization of transferrin is primarily mediated by transferrin receptor 1, and once internalized, is sorted to early and recycling endosomes [72],[73], we observed colocalization of 34.8% and 24.5% of Alexafluor 488-transferrin with EEA1 (Figure 11A and E, and Video S16) and Rab11 (Figure 11B and E, and Videos S17 and S18), respectively. Significantly, as much as 8.6% of transferrin was colocalized with both EEA1 and actin filaments, and as much as 3.5% of transferrin was colocalized with both Rab11 and actin filaments (Figure 11F), suggesting an active role of actin cytoskeleton in these endosomal steps. We also observed colocalization of 2.0% of the Alexafluor 488-transferrin with Rab7, a late endosome marker (Figure 11C and E, and Video S19), which is consistent with the observation that a small portion of transferrin is internalized through transferrin receptor 2-mediated endocytosis and sorted to late endosome/lysosome [74]. In spite of the low ratio of association of Alexafluor 488-transferrin with Rab7, surprisingly, as much as 1.9% of the transferrin was colocalized with both Rab7 and actin filaments (Figure 11F), indicating that most of the transferrin-containing Rab7 late endosomes were associated with actin filaments. These results suggest that actin dynamics might be heavily involved in late endosomal sorting and/or trafficking. Interestingly, we observed colocalization of as much as 21.6% of the Alexafluor 488-transferrin with LAMP-1 (Figure 11D and E, and Video S20), which probably reflected the accumulation of transferrin in lysosomes over time. Accordingly, only 0.8% of the transferrin was colocalized with both LAMP-1 and actin filaments (Figure 11F), suggesting that once reaching the lysosomes, the transferrin-containing endosomes no longer require actin filaments for sorting and trafficking. Together, these results suggest that, in addition to ligand internalization, actin dynamics are also involved in several other steps of clathrin-mediated endocytosis including endosomal sorting and trafficking from the early endosome through the late endosome/lysosome in endothelial cells.

## Discussion

The objective of this study was to identify the cellular pathways and factors that are exploited by KSHV for its entry and trafficking in endothelial cells. We demonstrate here the utility of an assay based on immunostaining of viral capsids to visualize KSHV virions that have successfully entered the cells and docked at the nucleus. Previous studies relied on the expression viral or reporter genes as indicators for successful viral entry [44],[75]. However, even a single viral particle can potentially enter a cell, travel to the nucleus, inject viral DNA into the nucleus and initiate an infection. Thus, these methods are less sensitive and are confounded by factors that influence the post-entry and trafficking events. Our results derived from assessing the effects of actin cytoskeleton disrupting agents on KSHV entry and infection have clearly illustrated this point (Figures 7 and 8). While we detected an effect of these inhibitors on the internalization of viral particles, the effect on KSHV infectivity was MOI-dependent. Similar to other herpesviruses, KSHV might use more than one route to enter cells [39]. In this case, inhibition of one pathway might not affect viral entry via another pathway, resulting in a reduced number of viral particles entering the cells, but the reduction in viral/reporter gene expression and viral infectivity will not be detectable. In fact, it has been demonstrated that if one endocytic pathway is blocked, the cells can actually upregulate alternate endocytic routes [76]. In addition, extended exposure to chemical inhibitors might affect the expression of viral/reporter genes simply because they are toxic to the cell rather than affecting the entry or trafficking events. Furthermore, inhibition of the later steps of endocytosis or endosomal trafficking does not necessarily prevent viral attachment or early internalization. Finally, removal of the inhibitors will allow the virus to complete the infection process if the inhibition is reversible. Certainly, the use of multiple inhibitors with differing modes of action, in a range of concentrations, to target a single pathway could impart more validity to the results of these experiments.

The results of this study clearly demonstrate an essential role for actin dynamics in KSHV entry and trafficking in endothelial cells. Endothelial cells are a highly relevant cell type for investigating KSHV infection as the neoplastic component of KS lesions is primarily composed of KSHV-infected endothelial cells. Although our results apparently contradict those in other studies, which did not identify actin cytoskeleton as a requirement for viral entry and trafficking [44],[45], several explanations can account for the discrepancy. First of all, if only the total number of KSHV-infected cells (nuclei with at least one Orf65+ viral capsid) is quantified, actin-disrupting agents did not seem to prevent viral entry (Figure 7B, F and J). However, when the absolute number of viral particles docked per nucleus is quantified, the effect of actin disruption on viral entry becomes clear (Figure 7C, G and K). Consistent with these results, when the infection was performed at a low MOI, the effect of actin-disrupting agents on the total number of KSHV-infected cells was also demonstrated (Figure 8B). These results were confirmed by LANA-based infectivity assay (Figure 8C) albeit the assay could be affected by post-entry events as discussed above. The results from the experiments using inhibitors of actin regulators (Figure 9) including CdTB, an inhibitor of Rho GTPases, and wiskostatin, an inhibitor of N-WASP, and the observation of viral capsids closely associated with actin filaments (Figure 10) further confirmed this conclusion. In fact, these results are consistent with previous observations that RhoA GTPase is important for KSHV entry in HEK293 cells [47] and association of viral particles with microtubules in DMVEC [48]. While these studies have never established a role for actin dynamics in KSHV entry and trafficking, they, nevertheless, support an essential role of Rho GTPases in KSHV entry and trafficking. Together, these observations are consistent with KSHV induction of actin dynamics during KSHV infection of endothelial cells, which include the disruption of actin stress fibers and growth of cortical actin structures at the early stage of KSHV entry (<15 mpi), and reappearance of actin filaments as actin tails or spikes at the later stage (>15 mpi) (Figure 6). Secondly, the results reported here are from experiments that exclusively used endothelial cells as the target cells for KSHV infection, while previous studies mainly used fibroblasts and HEK293 cells [44]–[48]. Herpesviruses have been found to use different entry pathways for different types of target cells. For instance, HSV-1 infects some cells through plasma membrane fusion but other cell types through endocytosis [40]–[42]. Plasma membrane fusion may not require the participation of actin cytoskeleton for virus internalization, while endocytosis does. Importantly, while we have identified clathrin-mediated endocytosis as the major route of entry used by KSHV to infect endothelial cells, none of the inhibitors used was able to completely block viral entry, suggesting the existence of an alternate route of entry in endothelial cells. The existence of such an alternative route could obviously confound delineation of the role of cellular components such as the actin cytoskeleton in regulating KSHV entry and trafficking if inappropriate assays and controls are used.

Studies of influenza virus entry have demonstrated that viral entry occurring at the apical surface of cells requires actin, while viral entry from the basolateral side did not [15],[20]. This suggests that the greater degree of membrane curvature at the apical surface might necessitate actin dynamic activity to induce membrane invagination, while the basolateral side does not [17]–[19]. In mammalian cells, the actin cytoskeleton is critical for the maintenance of plasma membrane tension. Chemical disruption of the entire actin cytoskeleton will relieve membrane tension, which may in some cases facilitate the formation of membrane invaginations, and obviate the need for actin polymerization to drive this step of endocytosis [5],[17],[18],[77]. In addition to forcing membrane invagination, actin might participate in several other steps of the endocytic pathway, beginning with the aggregation of receptors into clathrin-coated pits, scission of the newly-formed clathrin-coated vesicle away from the plasma membrane, movement of the clathrin-coated vesicle towards the cell interior, and trafficking of the vesicle as it matures along the endocytic pathway. Vesicle trafficking at the cell cortex might be actin-dependent, prior to transfer of the vesicle to microtubules to complete the journey to the perinuclear region. In fact, depolymerization of microtubules did not prevent KSHV binding or entry, but did prevent the delivery of viral particles to the nucleus [45]. KSHV infection also activated the formin family member mDia2 [45]. mDia1 and 2 coordinate actin assembly at the cell cortex [78], stabilize microtubules independently of actin nucleating activity [79], and localize to endosomes [80].

While there is evidence suggesting the involvement of the actin cytoskeleton in the internalization of cargo and initial formation of endosomes during clathrin-mediated endocytosis in mammalian cells, information on its role in endosome sorting and trafficking is limited [5]. In our study, KSHV capsids were observed to colocalize with actin filaments as well as markers for early and recycling endosomes, and lysosome. The fact that KSHV capsids were associated with actin filaments at the early time points of infection until the docking of the capsids at the nucleus suggests that actin dynamics might be involved in multiple steps of internalization, and endosomal sorting and trafficking of viral particles in endothelial cells. In agreement with these results, we have also observed colocalization of transferrin with actin filaments as well as markers of early, recycling and late endosomes, and to a lesser degree, lysosomes, further supporting the involvement of actin dynamics in multiple endosomal steps of clathrin-mediated endocytosis in endothelial cells. Additional studies are required to delineate the specific mechanism by which actin dynamics mediate different steps of endosomal sorting and trafficking during clathrin-mediated endocytosis. In this context, KSHV can be considered as a useful tool for elucidating the detailed mechanism of a vital cellular pathway.

## Methods and Materials

### Cell culture, viral stock and viral infection

Early passage of HUVEC were obtained from Clonetics, Lonza, and maintained in complete EBM-2 culture media (Allendale, NJ). KSHV-infected BCP-1 cells isolated from peripheral blood mononuclear cells of a PEL patient [81] were maintained in culture in RPMI1640 containing 10% fetal bovine serum (FBS). To induce virus production, BCP-1 cells in log-phase were serum-starved overnight in RPMI1640. FBS was added back to the culture media to a final concentration of 10%, together with 12-*O*-tetradecanoyl-phorbol-13-acetate (TPA) at 30 ng/ml and sodium butyrate at 200 µM. At 2 day post-induction, the cells were washed and the media was replaced with fresh RPMI1640 containing 10% FBS without TPA or sodium butyrate. At 6 days post-induction, the BCP-1 cells were centrifuged at 1,000×g for 5 min, and the supernatant containing viral particles was collected, and either used immediately for infection or stored at 4°C.

For viral infection, supernatant from induced BCP-1 cells was centrifuged for 10 min at 10,000×g. The pellet containing infectious viral particles was resuspended in one-fourth of the original volume in EBM-2 media with or without chemical inhibitors. To infect the cells, HUVEC were seeded onto glass cover slips overnight to achieve 70–80% confluency. For assays using chemical inhibitors, cells were pretreated with the inhibitors in EBM-2 media for 1 h prior to infection. The cells were then inoculated with the virus preparation and incubated for the indicated times in the presence of the inhibitors, fixed in 2% paraformaldehyde and processed for immunostaining of viral capsids and cellular markers.

### Antibodies and chemical inhibitors

A monoclonal antibody isotype IgG2a (clone 6A) to KSHV small capsid protein (Orf65) was used to stain KSHV capsids [82]. A rabbit antibody to gB was a kind gift of Dr. Bala Chandran at the Rosalind Franklin University of Medicine an Science, Chicago, Illinois. A monoclonal antibody to β-actin was obtained from Sigma (St. Louis, MO). A rat monoclonal antibody to LANA, and rabbit antibodies to clathrin heavy chain, β-tubulin, EEA1, Rab11, Rab7 and LAMP-1 were purchased from Abcam (Cambridge, MA). Chemical inhibitors of endosomal acidification bafilomycin and monensin were purchased from Sigma. NH_4_Cl was obtained from Fisher Scientific (Pittsburgh, PA). Chemical inhibitors of clathrin assembly chlorpromazine and dextrose, and caveolae/lipid raft-mediated endocytosis filipin, nystatin, and MβCD were purchased from Sigma. Chemical disruptors of the actin cytoskeleton latrunculin A and cytochalasin D were from Sigma while jasplakinolide was from Calbiochem, EMD Chemicals, Inc. (Gibbstown, NJ). The chemical inhibitor of WASP wiskostatin and inhibitor of Rho GTPases CdTB were from Calbiochem. All chemical inhibitor stock solutions were prepared according to the manufacturer's directions. AlexaFluor 488-phalloidin, AlexaFluor 488-transferrin, AlexaFluor 488-CTB, secondary antibodies AlexaFluor 568 goat-anti-mouse IgG1, AlexaFluor 568 and 647 goat anti-mouse IgG2a, and AlexaFluor 488 goat anti-rabbit IgG were from Molecular Probes, Invitrogen (Carlsbad, CA). DAPI was from BioChemika Ultra, Sigma. PI labeling kit was obtained from Roche (Nutley, NJ).

### Microscopy

For quantification of entry and trafficking of viral particles to the nuclei, images were acquired using a Zeiss Axiovert 200 M epifluorescence microscope equipped with a 63× oil immersion objective (Carl Zeiss Microimaging Inc., Thornwood, NY). Images were acquired for 5 fields of view per coverslip to allow counting of Orf65+ capsids docked at nuclei. All experiments were performed in triplicate. Results are expressed as the mean±SD. For colocalization experiments, images were acquired with an Olympus FV1000 scanning confocal microscope equipped with a 60× objective, NA 1.42 (Olympus Life Science, Center Valley, PA). Z-stacks were acquired at 0.25 µm per slice by sequential scanning. Olympus FV1000 software was used to generate cross-sectional images and 3D-projection images (Olympus Life Science).

### Cytotoxicity assay

To examine the cytotoxicity of the inhibitors, HUVEC grown in 48 well plates were treated with the inhibitors at the indicated concentrations for the stated period of time. One hour prior to fixation, the cells were labeled with PI to identify nonviable cells. Cells were washed twice with PBS, fixed with 2% paraformaldehyde for 30 min, and counterstained with DAPI. The cells were visualized with a 20× objective using the Axiovert 200 M fluorescence microscope. The total number of cells and the corresponding PI-positive cells from 5 representative fields were counted, and calculated as PI+ cells (%). In all the experiments, cells left to air-dry for 20 min to induce cell death were used as positive controls.

### Statistical analysis

All results were expressed as the means±s.d..

## Supporting Information

Figure S1 Visualization of enveloped viral particles at the early and late time points of KSHV infection in endothelial cells. HUVEC were infected with KSHV and stained for actin cytoskeleton (green), gB (red), Orf65 (magenta), and nuclei (blue) at 5 mpi (A) and 4 hpi (B). Over 90% of the viral particles inside the cells were positive for both gB and Orf65 at the early stage of infection (A) while almost all the viral particles were only positive for Orf65 but not gB at the late stage of infection (B). All viral particles outside the cells were positive for gB and Orf65 at both early and late time points of infection (A and B).
(2.60 MB EPS)

Figure S2 Inhibitors of endosomal acidification do not have any effect on cell viability. HUVEC were treated with inhibitors of endosomal acidification at conditions described in Figure 2 and evaluated for viability by staining with PI and DAPI. (A) Monensin; (B) NH_4_Cl; and (C) Bafilomycin. (D) Cells left to air-dry for 20 min to induce cell death were used as positive controls.
(6.68 MB EPS)

Figure S3 Inhibitors of clathrin-mediated endocytosis have minimal effect on cell viability. HUVEC were treated with inhibitors of clathrin-mediated endocytosis at conditions described in Figure 3 and evaluated for viability by staining with PI and DAPI. (A) Dextrose and (B) Chlorpromazine. (C) Cells left to air-dry for 20 min to induce cell death were used as positive controls.
(4.16 MB EPS)

Figure S4 Actin cytoskeleton-disrupting agents do not have any effect on cell viability. HUVEC were treated with actin cytoskeleton-disrupting agents at conditions described in Figure 7 and evaluated for viability by staining with PI and DAPI. (A) Cytochalasin D; (B) Latrunculin A; and (C) Jasplakinolide. (D) Cells left to air-dry for 20 min to induce cell death were used as positive controls.
(5.38 MB EPS)

Figure S5 Inhibitors of actin cytoskeleton regulators have minimal effect on cell viability. HUVEC were treated with inhibitors of actin cytoskeleton regulators at conditions described in Figure 9, and evaluated for viability by staining with PI and DAPI. (A) CdTB; and (B) Wiskostatin. (C) Cells left to air-dry for 20 min to induce cell death were used as positive controls.
(3.00 MB EPS)

Video S1 An overview of a HUVEC infected by KSHV shown in a XY section. Cells were stained for Orf65+ viral particles (red), actin cytoskeleton (green), and nuclei (blue). Images were acquired with an Olympus FV 1000 scanning confocal microscope using a 60× oil immersion objective.
(1.71 MB AVI)

Video S2 A cross-section (YZ) view of the same HUVEC infected by KSHV shown in Video S1.
(0.28 MB AVI)

Video S3 A 3D-projection image of the cell nucleus of the same HUVEC infected by KSHV shown in Video S1.
(0.12 MB AVI)

Video S4 A cross-section of the same cell nucleus shown in Video S3.
(0.16 MB AVI)

Video S5 3D-projection images of colocalization of Orf65+ viral particles with clathrin.
(0.73 MB AVI)

Video S6 3D-projection images of colocalization of Orf65+ viral particles with clathrin.
(0.57 MB AVI)

Video S7 3D-projection images of colocalization of Orf65+ viral particles with clathrin.
(0.72 MB AVI)

Video S8 3D-projection images of colocalization of Orf65+ viral particles with clathrin.
(0.67 MB AVI)

Video S9 3D-projection images of colocalization of Orf65+ viral particles with Alexafluor 488-transferrin.
(0.72 MB AVI)

Video S10 3D-projection images of colocalization of Orf65+ viral particles with Alexafluor 488-transferrin.
(0.73 MB AVI)

Video S11 3D-projection images of colocalization of Orf65+ viral particles with Alexafluor 488-transferrin.
(0.74 MB AVI)

Video S12 3D-projection images of colocalization of Orf65+ viral particles with Alexafluor 488-transferrin.
(0.69 MB AVI)

Video S13 A 3D-projection image of colocalization of an Orf65+ viral particle with an actin filament and EEA1.
(0.63 MB AVI)

Video S14 A 3D-projection image of colocalization of Orf65+ viral particles with actin filaments and Rab11.
(0.64 MB AVI)

Video S15 A 3D-projection image of colocalization of Orf65+ viral particles with actin filaments and LAMP-1.
(0.72 MB AVI)

Video S16 A 3D-projection image of colocalization of Alexafluor 488-transferrin with actin filaments and EEA1.
(0.59 MB AVI)

Video S17 A 3D-projection image of colocalization of Alexafluor 488-transferrin with actin filaments and Rab11.
(0.61 MB AVI)

Video S18 A 3D-projection image of colocalization of Alexafluor 488-transferrin with actin filaments and Rab11.
(0.81 MB AVI)

Video S19 A 3D-projection image of colocalization of Alexafluor 488-transferrin with actin filaments and Rab7.
(1.21 MB AVI)

Video S20 A 3D-projection image of colocalization of Alexafluor 488-transferrin with actin filaments and LAMP-1.
(0.52 MB AVI)
